# Our Correspondence

**Published:** 1870-10

**Authors:** 


### Our Correspondence.

### SIX LETTERS AND A POETICAL EFFUSION.

WASHINGTON, D. C., July 31, 1870.

EDITOR BISTOURY :

I wrote to the Rev. Charles E. King, M. D., L. L. D., *etc.*, *etc.*, of Bible House, New York City, for his *free* prescription for consumption. I received in reply a long lithographed letter, giving me considerable advice upon a subject that did not interest me or touch upon my case in the least, and wound up by "*prayerfully* commending me to the care of the Great Physician." Accompanying this letter was a circular informing me that the *Reverend* gentleman had his medicine for sale, for the small sum of Five dollars a bottle, and also inclosed me a religious tract on the "Importance of Religion to Society." I consider this the meanest sort of swindling. Any man that will use the cloak of religion to cover up his mean, dirty swindling operations, ought to be kicked by every honest man. Won't you give him a kick with BISTOURY ? I inclose the documents.

C.

— C's Letter explains itself. No comment is necessary.

The Reverend (?) gentleman mentioned is one of the many swindlers that find safe lodgement in New York, and who conduct their swindling operations with wonderful success and profit to themselves. The religious dodge is a good one, and succeeds well with the ignorant and unsophisticated. We have shown up the Reverend gentleman before.

---

WYALUSING, Pa., July 8, 1870.

DR. T. S. UP DE GRAFF.

*Dear Sir:*—Inclosed you ought to find one dollar to pay for two years of the BISTOTRY, and if you are successful in your search, you will not I hope, be after me with your BISTOURY, for keen as is its edge, its inscisions might not be painless.

I do not now order my copy of the paper stopped, as I am always anxious to see what the next number contains and have not the temerity to attempt to stop the instrument when poised in your hands.

I wish to ask you one question, and that is:—

Is it a shame and a reproach to a wife that she is also a mother? From the conversation of some of my friends, I am led to infer that more than *two* should be apologised for. Should my mother consider her nine children a "Crown of glory," or a bane to her?

G. H. W.

---

— Your mother should be, as she doubtless is, proud of her family of *nine* children. It would be far better for society, the State and the health of our American woman, if they thought more of rearing large and healthful families of children, and less of how to prevent such an increase. Every woman should be proud of her children, and if she is a true, noble and refined woman, she will delight in her exhibition of nine or even more.

---

COLUMBUS, Geo., Aug. 20th, 1870

DR. UP DE GRAFF.

*Dear Sir:*—Sometimes since, on reception of your polite hint, I inclosed you one dollar, as I have not received a copy since, I fear that some officials of the "Best Government the world ever saw" has appropriated it. The BISTOURY is a welcome visitor, and with its flashes of wit is mixed much instruction and sound practical sense. I have but two faults to find with it, and those are the ones which the Old-fashioned North Carolina governor found with his drinks: not enough of them and too long between.

Yours truly, X. Y.

---

— Thanks Doctor, for your good opinion of BISTOURY. We will see that it greets you regularly. Perhaps some day we will be able to remove your objections and have our journal put in a monthly appearance.

---

— And here follows a poetical effusion—a sort of reply to an item which appeared in the January number of the BISTOURY, relative to the near approach of a magnetic light which philosophers say is being hurled from the sun to the earth, and is approaching us at the rate of three and a half million miles a month:—

### IN THE LAST BISTOURY

You say the sun is travelling
  Toward earth, perhaps 'tis so;
'Tis not so easy telling where
  The Heavenly bodies go.

Should the sun keep moving on,
  And earth grow warmer still,
That light keep on increasing yet,
  (Undoubtedly it will),

Then what must be conclusions
  Of men who keep up thinking;
Those men who always seek the truth,—
  From God's word often drinking.

That is one way to find these things,
  From magic fountain flowing,—
From whence full well, we all can tell
  All changes worth the knowing.

All signs I'm watching—watching well,—
  Prophetic words I study;
But 'tis too soon to tell the world—
  To them it would be muddy.

I should not be surprised at all
  If '70 should be bolder
In startling facts, and signs more rare,
  Ere Earth is one year older.

We'll take advice a little while,
  And see what Sol 's about,
And with the gassing, gazing world,
  We'll keep a good "look out."

And if it comes along a pace,
  And lighter grows its beams,
We'll tell by and by, the reasons why,
  And what the whole thing means.

T. S. C.

---

— The following letter, from a namesake, explains itself:—

WILLIAMSPORT, PA., Aug. 5, 1870.

FRIEND BISTOURY:

Seeing in your valued work, several articles on the tape worm, I am reminded of the comical description of a friend from the west of Massachusetts, of a general time in a village there, on the tape-worm question, a few years since: Some child, it seems, was troubled with intestinal disturbances, and felt as if it had *surrounded* an enemy. By some accident or other, the child ate a great number of pumpkin seeds, and the result was the excommunication of a *long* friend, in the shape of a tape-worm. This being noised about, the neighbors and children had similar internal distractions, fancied or apparent, which led to the wholesale use of pumpkin-seed tea as a remedy. The result was an army of martyrs. But understand me, the martyrs were not the people, but the worms.

The tape-worm question always amuses me since, as it recalls the wholesale slaughter of the innocents, and some laughable incidents connected therewith.

Yours, THADDEUS.

### UTERINE SUPPORTER.

A feeling of much interest is being manifested by Physicians throughout the country with regard to the utility of Dr. L. A. Babcock's Uterine Supporter. For the information of those concerned, we insert the following communications on that subject:—

"NEW CASTLE, PA., Aug. 25, 1870.

I commenced using Dr. Babcock's Uterine Supporter in the treatment of uterine displacements about eight months ago. The first case in which I used it was a case of procidentia. It succeeded so well that I tried it again and again, until I have now made use of twenty of them.

"The almost universal testimony is that they are invaluable. I often hear expressions concerning them like the following: "I could not do without it." "I would not do without it for a hundred dollars;" and one patient was relieved by it so much that she declared she would not part with it for five hundred dollars, provided she could not get another. It almost uniformly relieves the aching pains about the back and hips, in cases of prolapsus, enabling them to walk, ride, do housework, etc., with comparative ease. I have recently been treating the cervix uteri of the wife of a clergyman. The day before I applied a Supporter she rode three miles in a carriage. At the end of her journey she had to lie down immediately, so great was the suffering through her back and hips.

The next morning I gave her one of the Supporters, and that day she rode in a carriage twenty miles with scarcely any suffering at all. She stated to me that for several years she had been unable to be on her feet for an hour at a time without suffering a great deal with pain in her back; but that since using the Supporter she could be on her feet half a day doing housework, ironing, etc., with scarcely any suffering at all. I have been for several years somewhat extensively engaged in the treatment of female diseases, and I have tried all kinds of pessaries such as globe, disc, ring, horshoe, etc., and I must say that notwithstanding they sometimes gave temporary relief, yet from the fact that in most cases the patients could not remove, clense and replace them, and also that they must rest on the perineum, thus relaxing it; that in the end they did about as much harm as good. I am satisfied that the stem pessary is the only kind which should be used, and Dr. Babcock's is the best adapted, easiest worn, and most efficient of any that I have tried, and the wonder is that it had not been discovered long ago.

Very truly yours,
M. T. BARKER, M. D."

---

"LIBERTY HILL, VA., July 13, 1870.

DEAR DOCTOR:

"Your request of my testimony in regard to Babcock's Silver Uterine Supporter has been received. I can now give you the result of my experience. The first instrument produced such favorable results in a case of retroversion of the womb that I was led to try the second in a like case. Case first had been an invalid for three years; not able to sit up or walk without assistance; now walks in the house anywhere, up stairs, etc., out into her garden, sits in the buggy and rides ten to twenty miles; visits her friends with impunity.

Case second had been confined *strictly* to her bed for sixteen months; could not sit up long enough to have her bed dressed. She is now walking in and about the house, riding on horseback and in a buggy, without suffering pain.

Both of my patients had the usual distressing symptoms attending this disease, many of which have altogether subsided, and others, (I feel safe in saying) will, on the continued use of the instrument. I can, so far as my experience with the instrument goes, highly recommend it to the profession as the one we have always needed for the cure of displacements of the uterus.

Very truly yours,
T. L. PAINTER, M. D."

